# Effect of Coffee on Lipopolysaccharide-Induced Immortalized Human Oral Keratinocytes

**DOI:** 10.3390/foods11152199

**Published:** 2022-07-24

**Authors:** Jianan Song, Byunggook Kim, Oksu Kim, Ying Yang, Danyang Liu, Wenqi Fu, Guowu Ma, Young Kim, Okjoon Kim

**Affiliations:** 1 Department of Oral Pathology, School of Dentistry, Chonnam National University, Gwangju 61186, Korea; songjianan87@gmail.com (J.S.); dentistyang@wmu.edu.cn (Y.Y.); liudysunny@gmail.com (D.L.); fwq0369@163.com (W.F.); youngkim2017@jnu.ac.kr (Y.K.); 2 Department of Oral Medicine, School of Dentistry, Chonnam National University, Gwangju 61186, Korea; bkkimom@jnu.ac.kr; 3 Department of Periodontology, School of Dentistry, Chonnam National University, Gwangju 61186, Korea; periodrk@jnu.ac.kr; 4 Department of Oral and Maxillofacial Surgery, School of Stomatology, Dalian Medical University, Dalian 116044, China; mgw640242000@aliyun.com

**Keywords:** artificial coffee, chlorogenic acid, caffeine, AMPK pathway, Nrf2/HO-1 pathway, periodontitis

## Abstract

Periodontitis is a common inflammatory disease that is strongly influenced by dietary habits. Coffee is one of the most common dietary components; however, current research on the relationship between coffee consumption and periodontitis, as well as its underlying mechanisms, is limited. Based on a previous report, caffeine (CA) and chlorogenic acid (CGA) were formulated into artificial coffee (AC) for this experiment. Cell viability, prostaglandin E_2_ release, Western blotting, cellular reactive oxygen species (ROS) production, and NF-E2-related factor 2 (Nrf2) translocation analyses were performed to explore the effects of AC on lipopolysaccharide (LPS)-induced immortalized human oral keratinocytes (IHOKs) and elucidate their underlying mechanisms. AC pretreatment attenuated LPS-induced inflammatory mediator release, ROS production, and nuclear factor kappa B translocation in IHOKs. CA and CGA promoted AMP-activated protein kinase phosphorylation and down-regulated the nuclear factor-κB pathways to exert anti-inflammatory effects. Additionally, CGA promoted Nrf2 translocation and heme oxygenase-1 expression and showed anti-oxidative effects. Furthermore, AC, CA, and CGA components showed synergistic effects. Thus, we predict that coffee consumption may be beneficial for alleviating periodontitis. Moreover, the main coffee components CA and CGA seem to play a synergistic role in periodontitis.

## 1. Introduction

Coffee is one of the most consumed beverages worldwide, and its consumption continues to increase, with an estimated 500 billion cups consumed daily [1]. Owing to its widespread consumption, even small health effects can be important on a population scale. Coffee consumption has been suggested to be associated with many health conditions, with mixed views on whether it is beneficial or harmful to health [2,3].

Recent epidemiological evidence suggests that coffee consumption decreases the risk of cardiovascular disease [4], mortality [5], chronic liver disease [6], type 2 diabetes [7], Parkinson’s disease [8], and some specific cancers [9,10,11]. One hypothetical explanation for the relationships between coffee consumption and positive health outcomes is that the components of coffee, such as polyphenols, have immunomodulatory and anti-inflammatory properties [12]. However, excessive coffee consumption can lead to adverse effects such as increased blood pressure, insomnia, nausea, diarrhea, polyuria, and mineral deficiencies [13,14,15].

Periodontitis is a common chronic inflammatory disease that leads to connective tissue degradation, irreversible alveolar bone destruction, and tooth loss [16,17]. Furthermore, it causes local damage to the mouth, and is also associated with systemic diseases [18]. Periodontitis has been shown to be strongly influenced by dietary habits [19]. Coffee is a common dietary component of daily life and some studies have shown that coffee intake is detrimental to periodontal health. For example, high caffeine or coffee intake may be associated with a risk of fractures and periodontal disease [20]. Daily consumption of coffee was also shown to delay the alveolar bone reparative process after tooth extraction [21]. In contrast, some studies have concluded that coffee consumption has no deleterious effects on periodontitis and is independently associated with the incidence of tooth loss [22]. A Japanese cross-sectional study found that coffee consumption during the maintenance phase of periodontal treatment reduced the prevalence of severe periodontitis [23]. Regardless, many unknowns remain regarding the effects of coffee consumption on periodontitis.

The current experimental study on the relationship between coffee consumption and periodontitis used coffee extracts or individual ingredients. However, coffee is a complex mixture and different types of coffee can have different compositions and biological effects [24]. This may be the reason for the inconsistent results of current coffee research. Previous reports have shown that the main components of coffee are caffeine and chlorogenic acid, which can be absorbed by the body and are present in stable amounts in the five most widely consumed coffees, except for decaffeinated coffee [25,26]. In the present experiment, we prepared artificial coffee (AC) using caffeine (CA) and chlorogenic acid (CGA) following the reported method. The CA and CGA content in one cup of AC for cells was equivalent to one cup of medium roast coffee (150 mL) for a human adult [25]. We explored the effects of CA and CGA, the main bioactive components of coffee, on lipopolysaccharide (LPS)-induced immortalized human oral keratinocytes (IHOKs).

## 2. Material and Methods

### 2.1. Chemicals and Antibodies

Caffeine and chlorogenic acid were obtained from Sigma-Aldrich (St. Louis, MO, USA) and LPS (from *Porphyromonas* *gingivalis*) was obtained from InvivoGen (San Diego, CA, USA). Specific antibodies against GAPDH and Nrf2 were purchased from Santa Cruz (Santa Cruz, CA, USA). Antibodies for NF-kB (P65), p-NF-kB (p-P65), COX-2, AMPK, p-AMPK, HO-1, and histone 3 were purchased from Cell Signaling Technology (Danvers, MA, USA).

### 2.2. Preparation of AC

Lee et al. reported that the primary constituents of dialyzed coffee extract are ~2 mM CA and ~1 mM CGA, and the CA and CGA content in 300 mL of dialyzed coffee extract is equivalent to 1 cup of medium-roasted coffee extract (150 mL) for a human adult (mean 60 kg, 59.4 L) [24]. Based on this information, 2 mM CA and 1 mM CGA were combined to formulate AC; the mixture was then sterilized by filtering it through a 0.2 µm-pore-size membrane filter (Millipore Corporation, Bedford, MA, USA) and immediately preserved at −70 °C until use. Therefore, 300 mL of AC for a human adult (i.e., 1 cup) was equivalent to 0.25 mL in 50 mL of culture medium for IHOKs (1 cup of AC contains 60 µmol CA + 30 µmol CGA, equivalent to the contents of 1 cup of coffee).

### 2.3. Cell Culture

IHOKs were cultured in Dulbecco’s modified Eagle’s medium mixed with Ham’s Nutrient Mixture F-12 at a ratio of 3:1 (Gibco, Grand Island, NY, USA), supplemented with 10% fetal bovine serum (JR Scientific, Woodland, CA, USA) and 100 U/mL penicillin/streptomycin (Welgene, Gyeongsan, Korea) at 37 °C in a humid 5% CO_2_ atmosphere. At 60% confluence, the cells were pretreated with AC, CGA, or CA at the indicated concentration for 24 h and then exposed to LPS (1 µg/mL) for a further 24 h. To inhibit the AMPK or Nrf2 pathway, IHOKs were pretreated with the AMPK inhibitor, compound C, or the Nrf2 inhibitor, ML385, for 2 h. The cells were grouped as follows and treated accordingly: control group, no treatment; LPS group, only LPS treatment; AC + LPS group, AC pretreatment and LPS treatment; CGA + LPS group, CGA pretreatment and LPS treatment; and CA + LPS group, CA pretreatment and LPS treatment.

### 2.4. Cell Viability Assay

The cytotoxicity of AC, CGA, and CA against IHOKs was evaluated using the MTT 3-(4,5-dimethylthiazol-2-yl)-2,5-diphenyltetrazolium bromide [MTT; Sigma-Aldrich]) method. Cells (5 × 10^3^ cells/well) were seeded into 96-well culture plates and treated with different concentrations of AC, CGA, or CA (0–100 cups) for 24 h. The culture medium with the MTT reagent was subsequently added to each well, and the cells were incubated for 4 h. The medium was then replaced with DMSO and the cells were incubated for 10 min. The absorbance was measured at 562 nm using a microplate reader (Bio-Rad, Hercules, CA, USA). The experiments were conducted in triplicate and the percentage of cell viability was determined as (absorbance value of the experimental group/absorbance value of the control group) × 100%. The concentrations of AC, CGA, and CA used in the subsequent experiments were selected based on the assay results. The same method was used to assess the viability of the IHOKs treated with LPS for 24 h.

### 2.5. PGE_2_ Release Assay

IHOKs were pretreated with or without AC, CGA, or CA according to the experimental design for 24 h and then treated with *LPS* (1 µg/mL) for 24 h. Conditioned supernatants were collected, and PGE_2_ levels were measured using a commercially available enzyme-linked immunosorbent assay (ELISA) kit (Parameter TM; R&D Systems, Minneapolis, MN, USA) according to the manufacturer’s instructions. The absorbance of PGE2 was determined using a microplate reader at 450 and 570 nm for wavelength correction.

### 2.6. Cellular Reactive Oxygen Species (ROS) Determination

Total intracellular reactive oxygen species (ROS) levels were determined using the 2′,7′-dichlorodihydrofluorescein diacetate (DCF-DA, Sigma-Aldrich) fluorescence method. IHOKs (5 × 10^4^ cells/well) were seeded in 6-well culture plates and pretreated with 10 cups of AC (containing 600 µmol CA and 300 µmol CGA), CGA (300 µmol), or CA (600 µmol) for 24 h, and subsequently treated with LPS (1 µg/mL) for 24 h. Cells were incubated with DCF-DA in phosphate-buffered saline (PBS) at room temperature (25 °C) for 15 min away from light. The solution was discarded, and the cells were rinsed twice with ice-cold PBS. The ROS formation index was measured through fluorescence microscopy (Lionheart FX, Bio-Tek, Winoski, VT, USA).

### 2.7. Immunofluorescence Staining

Nrf2 translocation was analyzed using immunofluorescence staining. IHOKs were seeded in 6-well culture plates at a density of 5 × 10^4^ cells/well. After treatments under the indicated conditions, cells were fixed with 4% paraformaldehyde in PBS for 20 min at room temperature and permeabilized with PBS containing 0.1% triton X-100 for 15 min. The cells were then blocked in 1% Bovine Serum Albumin (BSA)-PBS for 1 h at room temperature, and then incubated with anti-Nrf2 antibody diluted in 1% BSA-PBS (1:200) overnight at 4 °C. Subsequently, the cells were incubated with goat anti-rabbit IgG secondary antibodies (1:500) for 2 h at room temperature and washed thrice with 1% BSA-PBS. Nuclei were stained with diluted 2-(4-Amidinophenyl)-6-indolecarbamidine dihydrochloride (DAPI dihydrochloride) for 5 min. Fluorescence was captured using a confocal scanning microscope (Lionheart FX, Bio-Tek, Winoski, VT, USA).

### 2.8. Western Blot Analysis

The treated cells extracts were lysed in cold modified radioimmunoprecipitation assay (RIPA) buffer supplemented with a protease/phosphatase inhibitor cocktail and phenylmethanesulfonyl fluoride (PMSF) and quantified using the BCA Protein Assay Kit (Thermo Fisher Scientific, Rockford, IL, USA). Equal amounts of protein lysate aliquots were separated by 10% polyacrylamide gel electrophoresis and transferred onto a 0.45 µm polyvinylidene difluoride membrane (Millipore, Boston, MA, USA) in transfer buffer at 4 °C at 110 V for 1.5 h. The membrane was blocked with 5% nonfat skim milk in Tris-buffered saline with Tween (TBS-T) for 1 h at room temperature and incubated overnight at 4 °C with the following primary antibodies: AMPK (1:1000), p-AMPK (1:1000), NF-kB (P65; 1:1000), p- NF-kB (p-P65; 1:1000), COX-2 (1:500), Nrf2 (1:1000), HO-1 (1:1000), Histone 3 (1:1000), and GAPDH (1:2000). After washing with TBS-T, the membrane was incubated with the corresponding HRP conjugated anti-rabbit IgG or anti-mouse IgG secondary antibody (1:5000; Santa Cruz Biotechnology, Santa Cruz, CA, USA) for 2 h at room temperature. The protein bands were detected using an enhanced chemiluminescence system (Bio-Rad). Equal loading was measured using the GAPDH antibody to homogenize the amount of total protein.

### 2.9. Statistical Analysis

Data from independent experiments were expressed as the mean ± standard deviation, and analyzed using the IBM SPSS statistical software (version 22.0 SPSS Inc., Chicago, IL, USA). Statistical analysis of significance was performed using one-way ANOVA. The experiments were repeated at least twice for each assay and statistical significance was set at *p* < 0.05.

## 3. Results

### 3.1. Effects of AC on IHOK Viability

**Figure 1:**
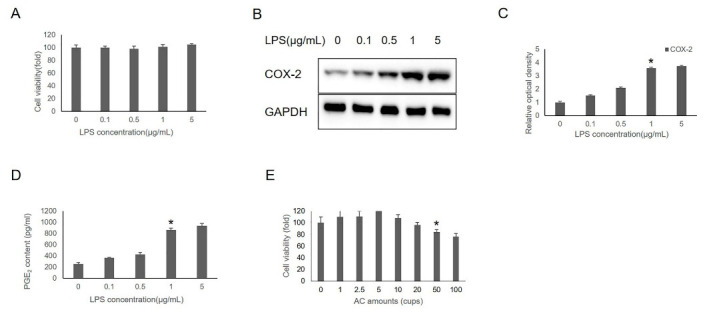
Effects of LPS and AC on IHOKs. MTT(3-(4,5-dimethylthiazol-2-yl)-2,5-diphenyltetrazolium bromide) assay was used to assess the cell viability of IHOKs after treatment with different LPS concentrations or AC amounts. (**A**) IHOKs were treated with 0–5 µg/mL LPS for 24 h. (**B**) COX-2 levels in 0–5 µg/mL LPS-induced IHOKs were detected by Western blot analysis. (**C**) Relative optical density was measured using Image J software. (**D**) The released PGE_2_ levels in 0–5 µg/mL LPS-induced IHOKs were detected using the prostaglandin E_2_ assay kit. (**E**) IHOKs were treated with 0–100 cups of AC for 24 h. * *p*-value < 0.05 vs. control.

To determine the LPS-treatment concentration, the cells were treated with 0–5 µg/mL of LPS for 24 h. The results show that treatment of cells with LPS (0–5 µg/mL) for 24 h had no effect on cell viability (Figure 1A), but significantly increased the level of COX-2 expression and PGE_2_ release at an LPS concentration ≥ 1 µg/mL, indicating the presence of inflammation (Figure 1B–D). The cytotoxic effects of AC on IHOKs were evaluated using the MTT assay. As shown in Figure 1E, the viability of IHOKs treated with AC showed no cytotoxicity at a concentration within 20 cups (containing 1.2 mmol CA + 0.6 mmol CGA, equivalent to the contents of 20 cups of coffee). However, when the concentration of AC reached 50 cups (containing 3 mmol CA + 1.5 mmol CGA equivalent to the contents of 50 cups of coffee), cell viability was significantly decreased compared to the control group. Therefore, subsequent experiments were performed using 1 µg/mL LPS for 24 h with or without 1–20 cups of AC (containing 60 µmol–1.2 mmol CA + 30 µmol–0.6 mmol CGA, equivalent to the contents of 1–20 cups of coffee) treatment.

### 3.2. Effects of AC on LPS-Induced Inflammation in IHOKs

**Figure 2:**
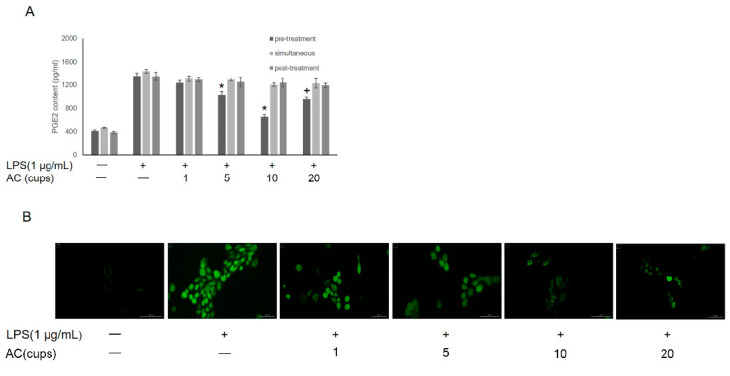
(**A**)The released PGE_2_ levels in pretreatment, simultaneous, and post-treatment groups were detected under treatment with 0–20 cups AC using the prostaglandin E_2_ assay kit. (**B**) The intracellular ROS formation under 0–20 cups AC pretreatment was detected with DCF-DA staining. * *p*-value < 0.05 vs. LPS treated control; + *p*-value < 0.05 vs. AC (10 cups). “-” represent untreated group; “+” represent treated with LPS group.

As shown in Figure 2A, PGE_2_ release was induced by LPS treatment and decreased by treatment with 1–10 cups of AC (containing 60 µmol–600 µmol CA + 30 µmol–300 µmol CGA equivalent to the contents of 1–10 cups of coffee), in a dose-dependent manner, an effect that was reversed with 20 cups of AC (containing 1.2 mmol CA + 0.6 mmol CGA, equivalent to the contents of 20 cups of coffee). Furthermore, the effect of AC on PGE_2_ release was stronger in the pre-treatment group than in the co-treatment and post-treatment groups. We also examined the effect of the same amounts of AC pretreatment on intracellular ROS production to evaluate its antioxidant activity (Figure 2B). Pretreatment with 1–10 cups of AC (containing 60 µmol–600 µmol CA + 30 µmol–300 µmol CGA, equivalent to the contents of 1–10 cups of coffee) resulted in a dose-dependent anti-oxidative and anti-inflammatory effect in LPS-induced IHOKs (Figure 2A,B). Therefore, to investigate the active ingredients and possible underlying mechanisms, subsequent experiments were performed involving pretreatment with 10 cups of AC (containing 600 µmol CA + 300 µmol CGA, equivalent to the contents of 10 cups of coffee), CGA (300 µmol), or CA (600 µmol).

### 3.3. Effects of CGA and CA on LPS-Induced IHOKs

**Figure 3:**
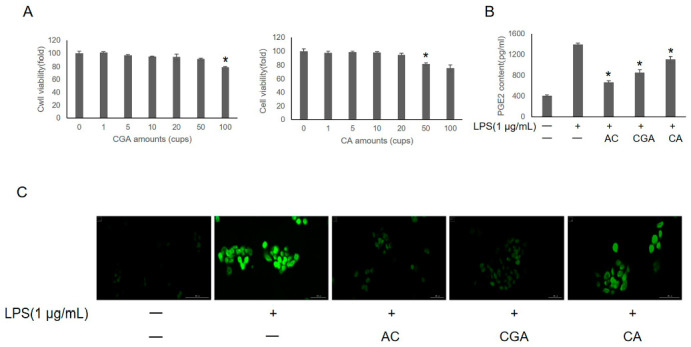
The effects of CGA and CA on IHOKs cell viability was analyzed by MTT. (**A**) IHOKs were treated with 0–100 cups of CGA or CA for 24 h. (**B**) The released PGE_2_ levels in only LPS and AC-LPS; CGA-LPS and CA-LPS groups were detected using the prostaglandin E_2_ assay kit. * *p*-value < 0.05. vs. LPS only treated control. (**C**) The intracellular ROS formation in only LPS and AC-LPS; CGA-LPS; CA-LPS groups were detected with DCF-DA staining. “-” represent untreated group; “+” represent treated with LPS group.

As the two main components of AC are CGA and CA, we determined the effects of these two constituents on cell viability. The CA-treated group showed reduced cell viability at amounts of 50 cups (3 mmol CA), consistent with the results of the AC (50 cups; 3 mmol CA + 1.5 mmol CGA) group, indicating that the cytotoxic effect of AC was mainly due to CA and not CGA (Figure 3A). The level of PGE_2_ increased after LPS treatment and significantly decreased with CGA or CA pretreatment (Figure 3B). Intracellular ROS production was measured using DCF-DA fluorescence. DCF fluorescence was significantly enhanced in the LPS-treated group and virtually eliminated in the AC- (CGA + CA) and CGA-pretreated groups (Figure 3C). However, CA exerted little effect on LPS-induced ROS scavenging in IHOKs.

### 3.4. AC, CGA, and CA Inhibit LPS-Induced Inflammation by Activating the AMPK Pathway

**Figure 4:**
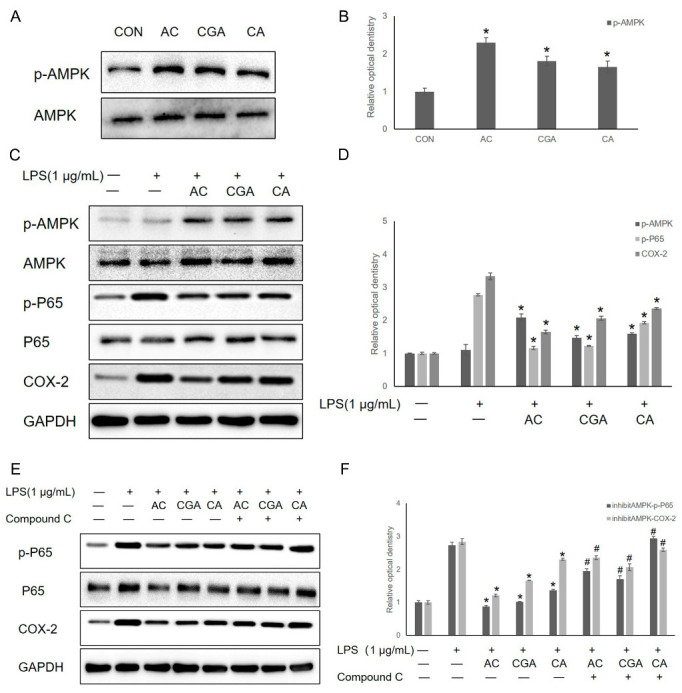
Western blot analysis. (**A**) The protein expression level of p-AMPK/AMPK in AC, GA, and CA pretreatment. (**B**) Relative optical density was measured using Image J software. (**C**) The expression levels of p-AMPK, AMPK, p-NF-κBp65, NF-κBp65, and COX-2 were determined by Western blot. (**D**) Relative optical density was measured using Image J software. (**E**) The expression levels of p-NF-κBp65, NF-κBp65, and COX-2 were determined using Western blot. (**F**) Relative optical density was measured using Image J software. * *p*-value < 0.05. vs. LPS only treated control. # *p*-value < 0.05. vs. compound C untreated control. “-” represent untreated group; “+” represent treated with LPS or compound C group.

Previous studies have suggested that the anti-inflammatory activity of coffee is associated with AMPK activation [1]. After treatment with AC, CGA, and CA, AMPK phosphorylation significantly increased (Figure 4A,B). To determine how the main components of coffee ameliorate periodontal inflammation, we examined AMPK activity and inflammation-associated protein expression in LPS-induced IHOKs. LPS exposure significantly enhanced P65 phosphorylation and COX-2 expression. However, pretreatment with AC markedly increase AMPK phosphorylation and decreased the p-P65 and COX-2 protein expression levels. Similar results were observed in both CA and CGA pretreatment groups (Figure 4C,D). Moreover, we pretreated the cells with compound C 2 h before AC (CGA + CA), CGA, or CA to inhibit AMPK activity. Western blot analysis results showed that compound C inhibited AMPK phosphorylation and the effect on LPS-induced p-P65 and COX-2 expression was reversed by compound C treatment (Figure 4E,F).

### 3.5. AC and CGA Inhibit LPS-Induced Inflammation by Activating the Nrf2/HO-1 Pathway

**Figure 5:**
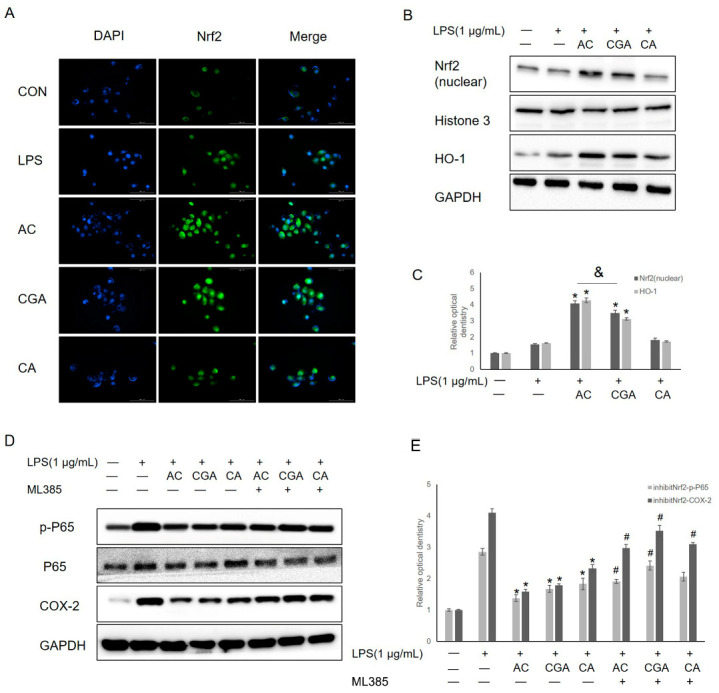
The effects of AC, GA, and CA on Nrf2 translocation. (**A**) The immunofluorescence staining was used to observe Nrf2 location via green fluorescence. 4‘,6-diamidino-2-2phenylindole (DAPI) was used to show the location of nucleus via blue fluorescence. (**B**) Nuclear Nrf2 and HO-1 expression levels were determined by Western blot. (**C**) Relative optical density was measured using Image J software. (**D**) The expression levels of p-NF-κBp65, NF-κBp65, and COX-2 were determined by Western blot. (**E**) Relative optical density was measured using Image J software. * *p*-value < 0.05. vs. LPS only treated control. # *p*-value < 0.05. vs. ML385 untreated control. & *p*-value < 0.05 (AC vs. CGA). “-” represent untreated group; “+” represent treated with LPS or ML385 group.

Immunofluorescence staining and Western blot analysis were used to explore whether CGA and CA affected the expression of the antioxidant marker protein HO-1 and its transcriptional factor Nrf2. IHOKs were pretreated with AC (CGA + CA), CGA, or CA for 24 h and then treated with LPS for 24 h. In the control group, Nrf2 was located in the cytoplasm, and nuclear Nrf2 staining was slightly more pronounced after treatment in the LPS only and CA-LPS groups (Figure 5A). Interestingly, nuclear translocation and increased expression of Nrf2 were noted after AC (CGA + CA) and CGA pretreatment, and its fluorescence intensity was the highest in the AC (CGA + CA)-LPS group (Figure 5A). These results indicate that pretreatment with CA exerted no effect on Nrf2 translocation, but did enhance the effect of CGA. To confirm these results, nuclear Nrf2 and HO-1 expression levels were measured using Western blot analysis. Similar to the results shown in Figure 5B, upon exposure to LPS, the nuclear Nrf2 and HO-1 expression levels increased slightly after treatment in the LPS-only and CA-LPS groups(Figure 5B,C). Indeed, the expression of nuclear Nrf2 and HO-1 significantly increased after AC (CGA + CA) and CGA pretreatment, with the highest levels being observed in the AC (CGA + CA)-LPS group (Figure 5B,C). The results indicated that pretreatment with CA alone exerted no effect on nuclear Nrf2 and HO-1 expression, but it enhanced the effect CGA on the levels of these proteins. To further clarify the relationship between Nrf2 translocation and inflammation, we used an Nrf2 inhibitor (ML385) in subsequent experiments. Based on previous reports [25], we pretreated the cells with ML385 2 h before AC (CGA + CA), CGA, or CA treatment to inhibit Nrf2 activity. Western blot analysis showed that the inhibition of Nrf2 reversed the protective effects of CGA on LPS-induced p-P65 and COX-2 expression. (Figure 5D,E).

## 4. Discussion

Coffee is the most popular beverage in the world. It is a complex mixture of more than 1500 components [26], and different coffee sources or brewing procedures can lead to differences in its composition and biological activity [27]. Previous studies have shown that the main constituents of coffee are CA and CGA, which occur in a 2:1 ratio [24]. In addition, all types of coffee, except decaffeinated coffee, contain almost equivalent amounts of CA and CGA [28]. Based on this information, we configured AC for this experiment, with the CA and CGA content in one cup of AC for IHOKs equivalent to one cup of medium-roast coffee [24], focusing on the effects of the main constituents of coffee on LPS-induced IHOKs and the associated mechanisms.

Many researchers have studied the effects of coffee and its major components on diseases in clinical and cell-based experiments. Coffee consumption reduces plasma biomarker concentrations of key metabolic and inflammatory pathway components in common chronic diseases [29], which could explain the association between coffee consumption and the reduced risk of several cancers, type 2 diabetes, ischemic stroke, Alzheimer’s disease, and Parkinson’s disease [30,31,32]. However, excessive coffee consumption can also be associated with adverse effects such as the constriction of blood vessels, increased blood pressure, and temporary increase in intraocular pressure [33,34]. The oral cavity is the first organ of the body to come in contact with coffee, and residual coffee components in the oral cavity after drinking may affect the health of periodontal tissues. Most studies have confirmed the benefits of coffee consumption in periodontal health [23,35,36,37,38]. In addition, CGA in coffee has a direct inhibitory effect on *P. gingivalis* [39]. However, a cross-sectional survey in Germany showed that moderate (3–6 cups/day) coffee consumption was not associated with periodontitis, whereas heavy coffee consumption (>7 cups/day) was significantly associated with periodontitis [35]. Studies on the effects of coffee and its main constituents, CA and CGA, on periodontitis are limited, and the results are controversial. Furthermore, interactions between these components cannot be ignored. Studies have shown that CA and CGA have synergistic effects on various processes, such as the inhibition of cyclooxygenase activity and the exacerbation of adipocyte degeneration [28,40]. The present study further investigated the effect of these two components on periodontitis and determined whether their mixture exerted a synergistic or antagonistic effect.

As the first line of epithelial defense, oral keratinized epithelial cells play a crucial role in preventing periodontal inflammatory diseases [41,42]. In the present study, we generated an inflammatory model of IHOKs using LPS, a common inflammatory stimulant [43]. Treatment of IHOKs with 1 µg/mL LPS did not affect cell viability but resulted in ROS generation, COX-2 expression, and PGE_2_ release. In contrast, pretreatment with AC significantly reduced the levels of these factors, indicating that AC possesses anti-inflammatory and antioxidant properties. Furthermore, we examined the effects of CGA and CA, at the same concentrations as those in AC, on the inflammatory mediators PGE_2_ and intercellular ROS. We found that the combination of CA and CGA attenuated LPS-induced PGE_2_ release and enhanced the anti-oxidative effect. The AMPK and Nrf2 signaling pathways are two common negative regulatory pathways in inflammation and oxidative stress [44,45]. Coffee extract activates the AMPK and Nrf2 pathways, which play important roles in metabolic regulation and cytoprotection [46,47,48]. However, the coffee components that activate AMPK and Nrf2 and whether these constituents have anti-inflammatory and anti-oxidative effects in periodontal inflammatory diseases are still largely unknown.

The AMPK and Nrf2 pathways were examined in the present study. Pretreatment with AC, CGA, or CA activated AMPK and inhibited NF-kB phosphorylation. Moreover, AC and CGA pretreatment significantly induced Nrf2 translocation to the nucleus and thus up-regulated HO-1 expression, whereas these anti-inflammatory effects were reversed by the addition of compound C (AMPK inhibitor) or ML385 (Nrf2 inhibitor). CA pretreatment exhibited no effect on Nrf2 translocation and HO-1 expression but enhanced the effect of CGA on these processes, showing a synergistic effect. Consistent with the results of previous studies, CA and CGA have also shown synergistic effects on other metabolic and inflammatory diseases [49,50,51]. Furthermore, CA has been shown to enhance the effectiveness of nonsteroidal anti-inflammatory drugs [52,53,54]. The dose of AC used in this study was seemingly high, as 10 cups of coffee per day must be consumed to achieve optimal protection. Previous studies have shown that three to four cups of coffee per day are the most effective in reducing the risk of various diseases [55], possibly owing to the synergistic effects of other coffee components. For example, the presence of flavonoids enhances the anti-inflammatory activity of caffeoylquinic acid [56]. In addition, digestion in the gastrointestinal tract affects the activities of coffee biocomponents, particularly polyphenols [57]. Studies have confirmed that the CGA content in coffee increases after digestion [58], which might enhance the anti-inflammatory effects of coffee.

In conclusion, the present study showed that CA and CGA promoted AMPK phosphorylation, and down-regulated the NF-κB pathway to exert anti-inflammatory effects. Additionally, CGA promoted Nrf2 translocation and HO-1 expression and showed anti-oxidative effects. Notably, AC, CA, and CGA components, exhibited synergistic effects on ROS scavenging and anti-inflammatory activities in LPS-induced IHOKs. Therefore, we suggest that coffee consumption may be beneficial for periodontitis, as the main coffee components CA and CGA seem to play a synergistic role in periodontitis.

## Data Availability

All data are contained in the article.
